# Six new bacterial species isolated from the phycosphere of marine macroalgae: a joint analysis based on taxonomy and polysaccharide utilization loci

**DOI:** 10.3389/fmicb.2025.1642517

**Published:** 2025-07-18

**Authors:** De-Chen Lu, Ying Yuan, Xin-Yun Tan, Le Liu, Jin-Hao Teng, Xue Cui, Tian-He Liu, Jing Zhang, Zong-Jun Du, Ming-Yi Wang

**Affiliations:** 1 Weihai Municipal Hospital, Weihai, China; 2 Marine College, Shandong University, Weihai, China

**Keywords:** core phycosphere families, *Flavobacteriaeae*, polysaccharide utilization loci, novel species, *Bacteroidota*

## Abstract

Marine macroalgae-associated *Bacteroidota* play crucial roles in global carbon cycling through polysaccharide degradation, yet their taxonomic and functional diversity remains understudied. Here, we describe six novel species (strains 3-376^T^, 4-2040^T^, 2-473A^T^, 4-528^T^, 4-911^T^ and 463^T^) within the families *Flavobacteriaceae*, *Crocinitomicaceae*, and *Cytophagaceae* isolated from macroalgal surfaces in the coastal area of Weihai, China. Metagenomic read recruitment and 16S rRNA abundance analyses demonstrated host-specific associations. Integrative taxonomic analyses, including phylogenomics (120 conserved proteins), 16S rRNA sequencing, and chemotaxonomy (e.g., MK-6 quinones, phosphatidylethanolamine lipids, and iso-C_15:0_ fatty acids), confirmed their novel status, with average amino acid identity (AAI), percentage of conserved proteins (POCP) distinguishing them from related taxa. Genomes (3.3–7.1 Mb; G + C 31.7–45.3%) revealed diverse polysaccharide utilization loci (PULs) targeting algal glycans like laminarin, alginate, and sulfated polymers (ulvan, chondroitin sulfate). *Cytophagaceae* 463^T^ harbored the richest CAZyme/PUL repertoire (131 CAZymes, 15 PULs), while *Crocinitomicaceae* 4-911^T^ lacked PULs, highlighting family-level specialization. This study expands the known diversity of core phycosphere *Bacteroidota*, linking PUL evolution to habitat specialization. The novel species’ distinct degradative capacities underscore their ecological roles in algal carbon processing and potential for biotechnological applications. Our integrated taxonomy-genomics approach advances understanding of microbial contributions to marine ecosystem dynamics.

## 1 Introduction

Marine macroalgae were foundational to coastal ecosystems, contributing approximately 1,521 teragrams of carbon (TgC) of annual biomass and driving global carbon cycling through photosynthetic carbon fixation (Krause-Jensen and Duarte, 2016). Their surfaces host the phycosphere, a dynamic microenvironment shaped by intimate interactions with bacteria, fungi, and other microbes over 1.6 billion years of co-evolution (Bengtson et al., 2017), and mostly belonging to yet-to-be described bacterial lineages. These phycosphere communities perform critical functions, including nutrient provisioning, stress acclimation, and morphological development of host algae, while also harboring pathogens or tissue-degrading commensals (Croft et al., 2005; Marshall et al., 2006; Martin et al., 2015; Dittami et al., 2016). Dominant phyla such as *Proteobacteria*, *Bacteroidota*, *Verrucomicrobiota*, *Planctomycetota*, and *Patescibacteria* mediate polysaccharide degradation and secondary metabolite production, processes central to carbon cycling and microbial community regulation (Lemay et al., 2021; Lu et al., 2023). The family *Flavobacteriaeae*, *Crocinitomicaceae* and *Cytophagaceae* were a monophyletic family within the phylum *Bacteroidota*. The *Flavobacteriaeae* and *Crocinitomicaceae* were the core families of marine macroalgae described by De-Chen (Lu et al., 2023). However, foundational knowledge on core phycosphere taxa, their genomic repertoires, and ecophysiological roles remains limited compared to terrestrial plant microbiomes.

Polysaccharide utilization locis (PULs) were genomic hallmarks of *Bacteroidota*, enabling efficient degradation and uptake of complex carbohydrates through coordinated systems of carbohydrate-active enzymes (CAZymes), sulfatases, and SusC/D transporter complexes (Martens et al., 2011). In marine environments, PULs have been linked to the breakdown of structurally unique algal polysaccharides—such as alginate, carrageenan, and ulvan—which feature sulfated, anionic motifs absent in terrestrial plants (Kappelmann et al., 2019; Lu et al., 2023) For example, *Polaribacter* clades in North Sea Spring blooms exhibit niche differentiation via specialized PULs targeting fucoidan, xylan, or laminarin, reflecting fine-scale ecological adaptation (Avcı et al., 2020). Yet, systematic inventories of PUL diversity in macroalgal-associated bacteria remain sparse, particularly for uncultivated or novel species. This gap limits understanding of how microbial communities process recalcitrant marine polysaccharides and contribute to carbon sequestration versus remineralization.

Strains of the *Bacteroidota* have been discovered in various marine environments, such as sea water (Avcı et al., 2020; Alejandre-Colomo et al., 2021), plankton (Avcı et al., 2020), marine sediment (López-Sánchez et al., 2024; Sun et al., 2025), phycosphere of algae or plants (Lu et al., 2023). The capacity to degrade various plant polysaccharides has been well studied in human gut *Bacteroidota* (Martens et al., 2011), and in some marine *Bacteroidota* targeting algal polysaccharides, e.g., alginate (Avcı et al., 2020), laminarin (Avcı et al., 2020), and carrageenan laminarin (Avcı et al., 2020). By efficiently degrading and utilizing complex polysaccharides, marine bacteria play a pivotal role in the global carbon cycle, facilitating the recycling of organic carbon in both terrestrial and aquatic ecosystems. Many members of *Bacteroidota*, including marine representatives of the family *Flavobacteriaeae* and *Crocinitomicaceae*, were specialized on polysaccharide degradation. PUL analysis of epiphytic bacteria co-occurring with macroalgae could serve as an alternative starting point to advance insight into the structures of marine polysaccharides and to understand their microbial decomposition. Current challenges in phycosphere research include resolving the structural complexity of algal polysaccharides and linking microbial genomic potential to *in situ* metabolic activity. While structural elucidation of macroalgal glycans has progressed, precise configurations often remain unresolved due to methodological hurdles (Sichert et al., 2020). Similarly, metaproteomic studies reveal temporal shifts in SusC/D expression during phytoplankton blooms, indicating dynamic utilization of substrates like laminarin and xylan (Kappelmann et al., 2019). However, these insights were largely confined to well-characterized clades, leaving understudied lineages-such as novel *Flavobacteriia*-poorly contextualized within broader carbon cycling frameworks.

During our survey of polysaccharide utilization capacity of phycosphere microbiota from 2018 to 2019 in the costal of Weihai, we identified five novel species and one novel genus (3-376^T^, 4-2040^T^, 2-473A^T^, 4-528^T^, 4-911^T^ and 463^T^) belonging to the family *Flavobacteriaeae*, *Crocinitomicaceae* and *Cytophagaceae*. We (i) identified the six distinct *Bacteroidota* novel species using phylogenetic analyses, (ii) quantified their abundances using 16 s rRNA gene and metagenome read recruitment, (iii) compared metagenome-assembled genomes (MAGs) for all of the six species, and (iv) conducted comparative analysis of PULs from six novel species by reanalyzing genome data. Furthermore, PUL annotation using high-quality representative genomes/MAGs were performed for the purpose of ecogenomic characterization. We tested whether difference species exhibit differences in their PUL repertoires that might explain their potential for polysaccharide utilization.

## 2 Methods

### 2.1 Isolation and culture condition

To investigate the ability of bacteria from the phylum *Bacteroidota* to degrade marine polysaccharides, marine 48 macroalgae, 12 seawater and 12 sediment samples were collected from a coastal area in Weihai, China, and a total of 5,527 isolates and 1,687 isolates belonging to the *Bacteroidota* (Lu et al., 2023). Sample dilution, spreading, culture conditions, and strain preservation refer to Lu et al. (2023). 91 species collected from LPSN and related genome download from NCBI. We have selected five novel species and one novel genus belonging to the core families of marine macroalgae.

### 2.2 Physiological characterization

Phenotypic characterizations were performed during the exponential period of growth. Accurate verification of negative or positive results was performed according to the bioMérieux Gram staining kit manual. Colony surface morphology was observed using a light microscope (E600, Nikon) and a scanning electron microscope (FEI Nanonova SEM450). The range of temperature for growth was tested at the following temperatures: 4, 15, 20, 25, 28, 30, 33, 35, 37 and 40°C. The pH was modulated to pH 5.5–9.5 (0.5 pH units apart) by adding the same densities and dissimilar pH value buffers in marine broth 2,216 (MB; Becton Dickinson) to detect the effect of pH on cellular development. Bacteria were cultivated in derivative MA supplemented with artificial seawater without NaCl as the basal medium, and their salinity and salt tolerance were evaluated. The salt gradient of the medium was set at 0–10.0% NaCl (0.5% intervals). All experiments were repeated three times unless otherwise noted. Testing for catalase activity with a 3% H_2_O_2_ solution and oxidase activity using an oxidase test kit from bioMérieux. Anaerobic growth was assessed over 15 days at 30°C in an anaerobic bag, on modified MA medium with or without 0.1% (w/v) NaNO_3_. Hydrolytic capabilities for agar, starch, alginate, casein, CM-cellulose, DNA, and lipases (Tweens 20, 40, 60, and 80) were evaluated according to the CLSI (2021) method. API 20NE tests were conducted in accordance with the manufacturer’s instructions, except the salinity was adjusted to 3% and performed with three biological replicates and two reference strains each time.

### 2.3 Chemotaxonomic characterization

During the late exponential phase of growth, cell masses from strain 3-376^T^, 4-2040^T^, 2-473A^T^, 4-528^T^, 4-911^T^ and 463^T^, and control strains were harvested from cultures grown on MA under ideal conditions to analyze fatty acids, respiratory quinones, and polar lipids. The harvested cells were freeze-dried, and from these dried cell masses, 10 mg was used to extract fatty acids. These extracts were then analyzed with an Agilent gas chromatograph (model 6,890 N), and the fatty acids were identified using the Microbial Identification System (MIDI database: TSBA40) (Sasser, 1990). For the analysis of respiratory quinones, 300 mg of the freeze-dried biomasses underwent purification as per the methods described by Minnikin et al. (1984) and were subsequently analyzed using high-performance liquid chromatography (HPLC). Polar lipids were extracted using varying ratios of a chloroform/methanol/water solution and analyzed via two-dimensional silica thin-layer chromatography. The identification of total polar lipids was performed using specific detection reagents, following established protocols by Minnikin et al. (1984).

### 2.4 Genome extraction, sequencing, 16S rRNA gene phylogenetic analysis, genomic analysis and abundance analysis of MAGs and draft genome

Genomic DNA of the six strains were extracted and purified using a bacteria genomic DNA kit (Takara). Strains sequencing were performed by Beijing Novogene Biotechnology (Beijing, China) on a NovaSeq (Illumina, San Diego, CA, USA) with 150 bp PE reads at ≥ 100 × coverage. Reads were quality-filtered and assembled with SPAdes v3.9.1 (Bankevich et al., 2012) (−careful –cov-cutoff) with k-mer sizes from 27 to 127 bp and a minimum scaffold length of 200 bp. The complete 16S rRNA gene sequences of strain 3-376^T^, 4-2040^T^, 2-473A^T^, 4-528^T^, 4-911^T^ and 463^T^, were extracted from the draft genomes by ContEst16S^1^. The returned 16S rRNA gene sequence was submitted to GenBank database and the sequences were analyzed using BLAST^2^ and EzBiocloud^3^ to determine their approximate taxonomic affiliations. Phylogenetic trees were reconstructed by the neighbor-joining (NJ) methods with MEGA 11 (Tamura et al., 2021).

Genes were predicted using Prodigal v2.6.3 (Hyatt et al., 2010) and annotated with Prokka (Seemann, 2014). The specific function of gene CAZymes-rich gene in PULs were searched in Carbohydrate Active Enzymes database^4^. The prediction and annotation of CAZymes-rich and PULs gene clusters were using dbCAN2 to find these clusters (Zhang et al., 2018). The DNA G + C content was calculated based on the whole genome sequence and the metabolic pathways were analyzed in detail employing KEGG’s KofamKOALA server (Kanehisa et al., 2016). Other relevant information for genome evaluation was obtained using CheckM (Parks et al., 2015). The phylogenetic relationship based on genome sequences was analyzed via GTDB-Tk (Chaumeil et al., 2020) and use 120 ubiquitous single-copy proteins. Pairwise average nucleotide identities (ANI) were calculated using the pyani Python3 module^5^, average amino acid identity (AAI) (Rodriguez-R and Konstantinidis, 2014) and percentage of conserved proteins (POCP) (Qin et al., 2014) were calculated by using an AAI and POCP calculator^6^. Five additional MAGs from epiphytic were part of BioProject PRJEB50838, and have been published and named previously (Lu et al., 2023). In order to determine MAGs and genomes abundance, metagenomic raw reads were mapped to MAGs using BBMap (minid = 99). Reads per kilobase per million (RPKM) was calculated based on MAG length and number of reads mapped.

## 3 Results

### 3.1 Characterization of the isolate collection

The 5,527 isolates were assigned to different taxonomic groups, but 1,687 belong to the phyla *Bacteroidota* were predominantly isolated. Five isolates could be assigned to the core phycosphere family *Flavobacteriaeae* and *Crocinitomicaceae* and presumptively identified as core phycosphere family, both of which were well-documented inhabitants of the phycosphere across diverse macroalgae species. Four isolates were novel species assigned to the family *Flavobacteriaeae*, one novel genus assigned to the family *Cytophagaceae* and one novel specie assigned to the family *Crocinitomicaceae*. These six isolates were selected for an in-depth taxonomic characterization because of their low 16S rRNA gene sequence similarities to already described species. Furthermore, a total of 5 MAGs belonging to *Maribacter algarum* 4-528^T^, *Tamlana algarum* 4-2040^T^ and *Aurantiphycus algarum* 463^T^ were used for reconstructing the phylogenomic tree (Supplementary Table S5).

### 3.2 Physiological, phenotypic and chemotaxonomic characteristics of the six selected strains

The six isolates were phenotypically characterized and classified based on their 16S rRNA gene sequences within the phyla *Bacteroidota*, representing five novel species and one novel genus. All strains grew within a temperature range of 20°C to 35°C, and no growth was seen below 10°C or above 45°C. All isolates were Gram-stain-negative. Additional phenotypic traits of the strains were presented in Supplementary Table S1, which highlights the comparative phenotypic distinction between the four isolates and adjacent strains of *Flavobacteriaeae*, including their ability to hydrolyze macromolecular compounds and enzymatic activity. The other two strains belong to *Cytophagaceae* and *Crocinitomicaceae* ability to hydrolyze macromolecular compounds and enzymatic activity. Physiologically, the strains exhibit mesophilic growth, with temperature optima between 25 and 33°C and broad pH tolerance (5.5–9.0). NaCl tolerance ranges varied significantly: *Maribacter algarum* 4-528^T^ displayed the widest salinity adaptation (0–10% NaCl), while *Brumimicrobium ulvae* 4-911^T^ thrived optimally at 6% NaCl, suggesting halotolerant tendencies. Metabolically, Tween utilization was widespread, with strain 3-376^T^ capable of hydrolyzing all Tweens (20–80), whereas strain 463^T^ showed no Tween activity. Starch hydrolysis was observed in *Eudoraea algarum* 2-473A^T^, *Maribacter algarum* 4-528^T^, and strain 463^T^, while cellulose degradation was exclusive to *Brumimicrobium ulvae* 4-911^T^. Proteolytic activity (casein hydrolysis) was detected in strains 2-473A^T^, 4-911^T^, and 463^T^. Oxidase and catalase activities were nearly universal, except in strain 463^T^ (catalase-negative) and *Eudoraea algarum* 4-528^T^ (oxidase-negative). Nitrate reduction occurred in four strains, with *Brumimicrobium ulvae* 4-911^T^ being a notable exception. All strains consistently tested negative for L-tryptophan degradation, D-glucose fermentation, arginine dihydrolase activity, urease production, and citrate utilization, as well as for the metabolism of carbohydrates such as glucose, arabinose, mannose, mannitol, N-acetyl-glucosamine, maltose, and potassium gluconate (Supplementary Table S1). Similarly, no activity was observed for acid production from capric, adipic, malic, or phenylacetic acid across all isolates. These uniform negative results suggest a shared metabolic limitation in these pathways among the tested strains. Key differentiating features emerged from tests for *β*-glucosidase, gelatin liquefaction, and β-galactosidase activity. Strain *Ulvibacter algarum* 3-376^T^ uniquely exhibited a negative result for β-glucosidase, whereas all other strains, including *Tamlana algarum* 4-2040^T^, *Eudoraea algarum* 2-473A^T^, *Maribacter algarum* 4-528^T^, *Brumimicrobium ulvae* 4-911^T^, and *Aurantiphycus algarum* 463^T^, tested positive (Supplementary Table S1). Gelatin liquefaction was observed exclusively in *Maribacter algarum* 4-528^T^, *Brumimicrobium ulvae* 4-911^T^, and Aurantiphycus algarum 463^T^, distinguishing them from the non-liquefying *Ulvibacter algarum* 3-376^T^, *Tamlana algarum* 4-2040^T^, and *Eudoraea algarum* 2-473A^T^. Notably, β-galactosidase activity was detected only in *Eudoraea algarum* 2-473A^T^, representing a unique trait among the isolates.

The chemotaxonomic features of the strains were consistent with those of the family *Flavobacteriaeae*, *Cytophagaceae* and *Crocinitomicaceae*. All strains contained MK-6 as the sole respiratory quinone. The polar lipid profile of strains 3-376^T^, 4-2040^T^, 2-473A^T^, 4-528^T^, 4-911^T^ and 463^T^ consisted of phosphatidylethanolamine. Except for *Maribacter algarum* 4-528^T^, the other five strains also contain AL, which belongs to the major polar lipids (Supplementary Figure S1; Supplementary Table S1). The major fatty acid constituents (>5%) of strains were iso-C_15:0_ and iso-C_17:0_ 3-OH. Cellular fatty acid compositions (%) derived from FAME analysis of all six strains were shown in Supplementary Table S2.

### 3.3 High-quality genome sequences of the *Bacteroidota* strains isolated from the macroalgal surface

**Figure 1:**
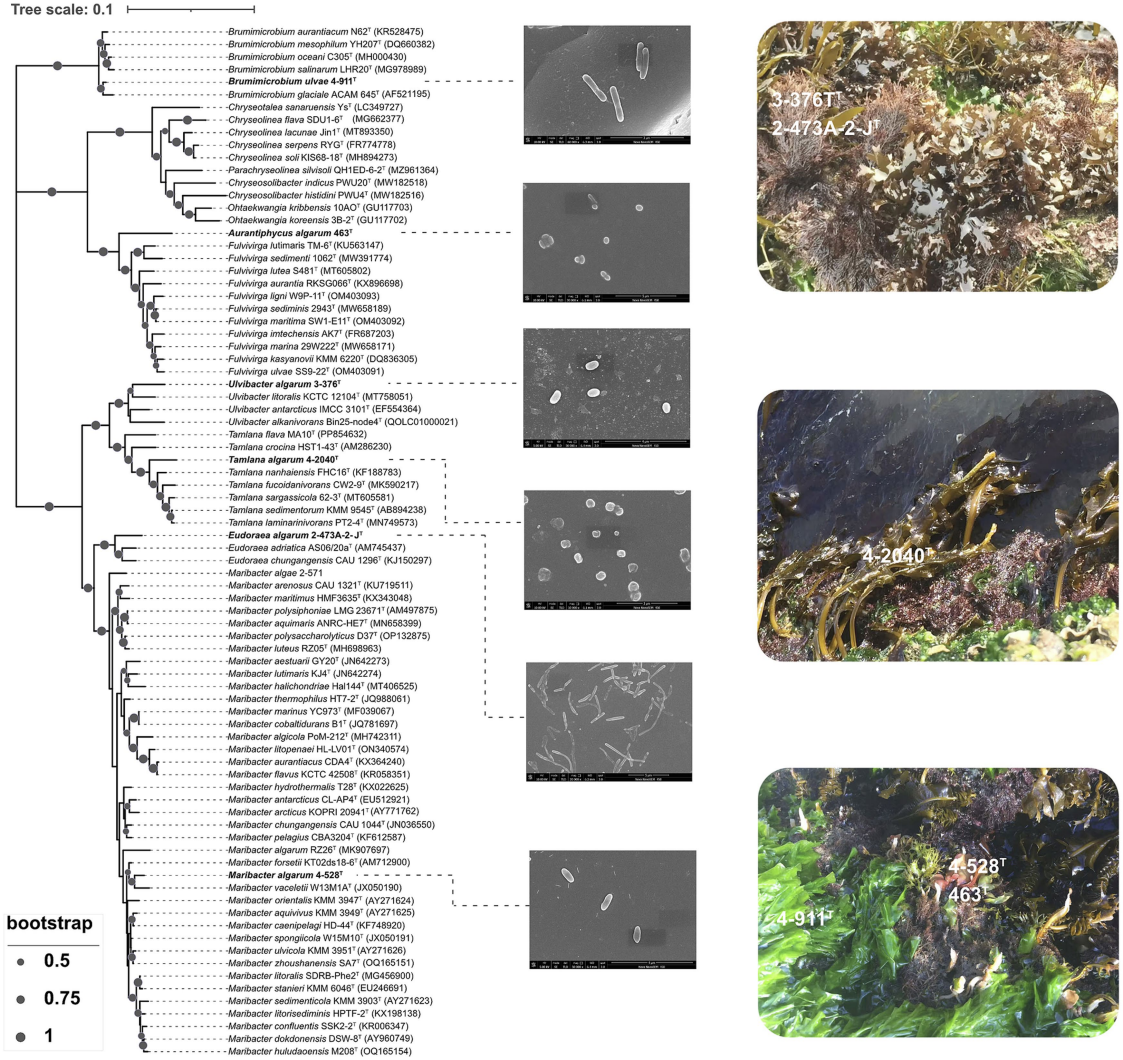
Neighbor-joining phylogenetic tree based on 16S rRNA gene sequences showing the relationship of strain 3-376^T^, 4-2040^T^, 2-473A^T^, 4-528^T^, 4-911^T^ and 463^T^. Bootstrap values (expressed as percentages of 1,000 replications) of >50% were shown at branching nodes. Bar 0.1 substitutions per nucleotide position. Scanning electron micrograph of cells of strain 3-376^T^, 4-2040^T^, 2-473A^T^, 4-528^T^, 4-911^T^ and 463^T^. Cells were grown on marine agar 2216 (MA) at 28.0°C for 3 days.

**Figure 2:**
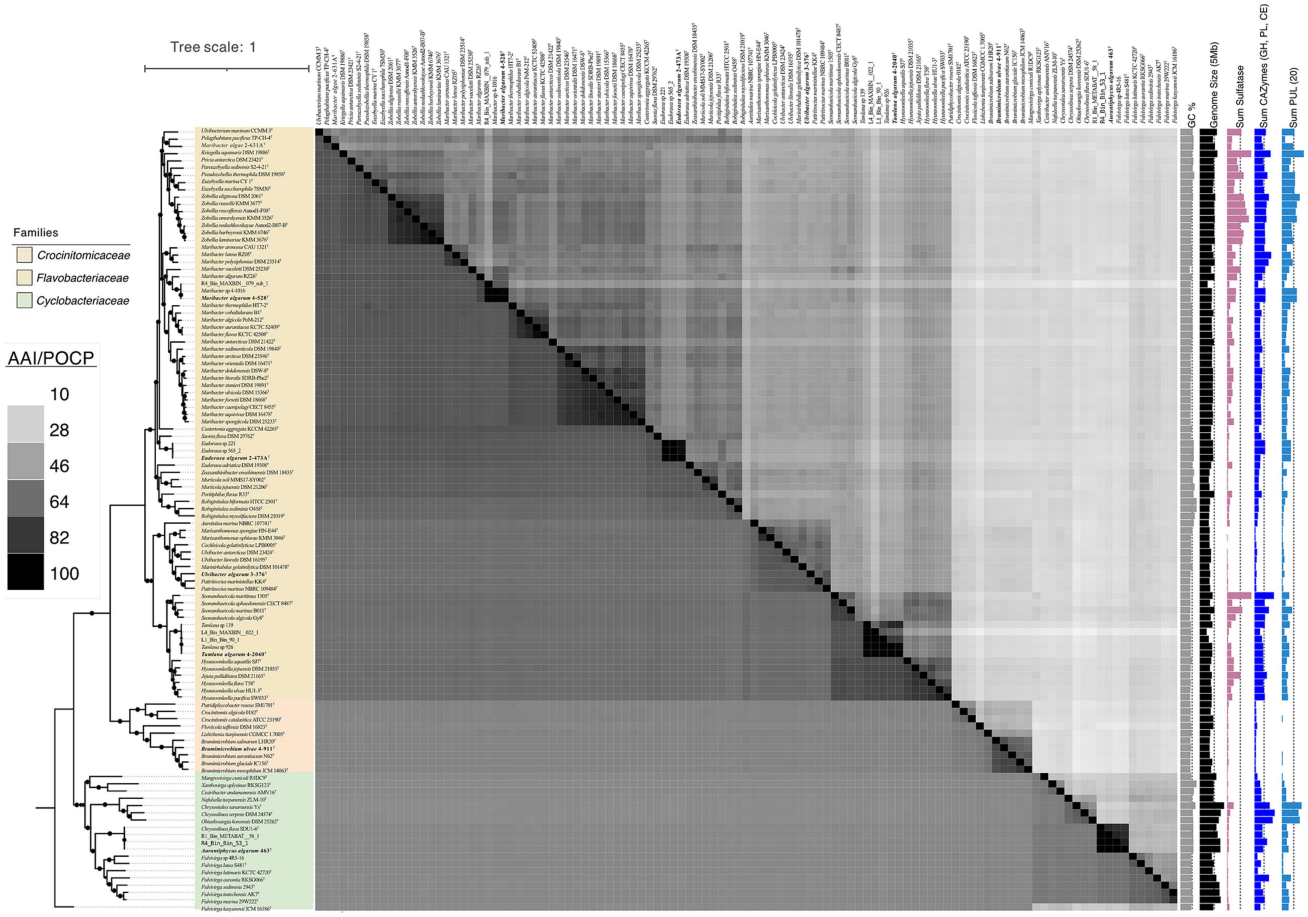
Phylogenetic tree of genomes based on the concatenated alignment of 120 ubiquitous single-copy proteins, highlighting the novel strains 3-376^T^, 4-2040^T^, 2-473A^T^, 4-528^T^, 4-911^T^ and 463^T^ within related taxa. The lower left part of the figure represents AAI values and the upper right part represents POCP values. Darker to lighter colors indicates higher to lower values. A darker color indicates a higher value. The scale bar represents 0.1 substitutions per amino acid position. The isolated habitats, genome sizes, and G + C content of the novel species and their related species were also displayed. Sum of PUL, CAZymes gene and Sulfatase genes of different bacterial species were represented by the bar graphs on the right.

The genome size of the strains was around 3.3–7.1 Mb with GC content ranging between 34.4 and 45.3%. *Brumimicrobium ulvae* 4-911^T^ (*Crocinitomicaceae*) and *Aurantiphycus algarum* 463^T^ (*Cytophagaceae*) represented the smallest and largest genomes of 3.3 Mb and 7.1 Mb, respectively, as well as the lowest and highest number of predicted genes with 2,912 and 5,780 (Supplementary Table S3). Four *Flavobacteriaeae* member showed a genome size average of 4.4 ± 0.9 Mb. In addition, *Ulvibacter algarum* 3-376^T^ showed the lowest G + C mol% content with 31.69%, and *Eudoraea algarum* 2-473A^T^ the highest with 45.2%. Phylogenetic analyses using amino acid sequences from the genomes, conducted with FastTree methods, showed that the five strains do not cluster together as a single group. However, 4-528^T^ show relatively closer clustering, forming a distinct lineage within the highest 16S rRNA gene similarity species (Figures 1, 2).

### 3.4 Phylogenetics, ANI, AAI clustering, phylogenomics, and population genomics

Phylogenetic reconstructions showed consistent topologies regardless of the sequences used to reconstruct them. Specifically, (i) the 16S rRNA genes (Figure 2), (ii) the concatenated sequences of 120 conserved single-copy orthologous genes (essential genes; Figure 1). The 16S rRNA gene sequences of strains 3-376^T^, 4-2040^T^, 2-473A^T^, 4-528^T^, 4-911^T^ and 463^T^ were obtained.

*Aurantiphycus algarum* was represented by strain 463^T^ and it had a 16S rRNA gene identity of 91.7% with its closest relative type strain of *Fulvivirga sedimenti*; *Eudoraea algarum* represented by 2-473A^T^ had a 16S rRNA gene identity of 95.3% with *Eudoraea chungangensis*; *Maribacter algarum* represented by strains 4-528^T^ had a 16S rRNA gene identity of 97.0% with *Maribacter vaceletii*; *Tamlana algarum* represented by 4-2040^T^ had a 16S rRNA gene identity of 95.8% with *Tamlana fucoidanivoran*; *Ulvibacter algarum* 3-376^T^ and *Brumimicrobium ulvae* 4-911^T^ had a mean 16S rRNA gene identity of 95.8, 97.6 and 97.3% with *Ulvibacter litoralis*, *Maribacter arenosus* and *Brumimicrobium aurantiacum*. The genome-based phylogenetic analyses were conducted (Figure 1). The tree indicates close relationships of strains 3-376^T^, 4-2040^T^, 2-473A^T^, 4-528^T^, 4-911^T^ and 463^T^ despite the relatively low level of average branch support. The inconsistencies between the phylogenetic trees based on the 16S rRNA gene sequences and the phylogenomic trees constructed from whole-genome sequence analyses reveal that 16S rRNA gene sequence analyses were insufficient to understand the phylogeny and evolution of the members of the *Bacteroidota*. Both the sequence similarities and phylogenetic relationships indicated that strains 3-376^T^, 4-2040^T^, 2-473A^T^, 4-528^T^, 4-911^T^ and 463^T^ represent five novel species and one novel genus of the *Bacteroidota*. The phylogenomics tree included three families. The *Crocinitomicaceae* was relatively small and contains 10 type strains. The family *Flavobacteriaeae*, however, was the largest and includes the most model species. It represents a highly diverse group in terms of genomic size and ecological diversity, with strains from various environments. Overall, this division provides valuable insights into the evolutionary, ecological, and genomic diversity among these strains, highlighting the differences in model species representation and the variability in genomic characteristics across difference families. A detailed summary of the overall genome-relatedness indices was provided in Supplementary Table S3.

The general features of the genomes were given in Supplementary Table S4. The ANI values between strain 3-376^T^, 4-2040^T^, 2-473A^T^, 4-528^T^, 4-911^T^ and 463^T^ with other strains of the genus were 74.72, 74.30, 71.95, 74.80, 75.97 and 68.98% (Supplementary Table S4), which were far below the standard ANI criteria for species identity (95.0–96.0%). The taxonomic classification of six novel strains 3-376^T^, 4-2040^T^, 2-473A^T^, 4-528^T^, 4-911^T^ and 463^T^ was rigorously validated through genomic metrics, including AAI and POCP (Supplementary Table S5). These analyses robustly support the delineation of five novel species within established genera and one novel genus. For strains *Ulvibacter algarum* 3-376^T^, 4-2040^T^ (Tamlana algarum), *Eudoraea algarum* 2-473A^T^, and Maribacter algarum 4-528^T^, AAI values ranged from 63.66 to 79.30%, well below the intra-genus threshold of 65–70%, while POCP values (45.61–59.63%) further confirmed their divergence from congeneric species. Notably, *Brumimicrobium ulvae* 4-911^T^ exhibited higher intra-genus POCP (68.36–70.21%) and AAI (75.88–77.60%), yet these values remained below species-level thresholds (>95%), supporting its classification as a novel species within *Brumimicrobium*. In contrast, strain 463^T^ displayed POCP values of 23.78–27.07% with members of *Fulvivirga* and *Chryseolinea*, far below the genus delineation threshold (>50%), alongside unique phenotypic and phylogenetic traits. Combined with the absence of close AAI comparators, these data unequivocally establish 463^T^ as the type strain of the novel genus *Aurantiphycus*. Collectively, the integration of AAI and POCP metrics underscores the taxonomic novelty of these strains, resolving their placement within the *Bacteroidota* phylum while highlighting the necessity of polyphasic approaches for robust microbial classification.

### 3.5 Habitat distribution analysis

**Figure 3:**
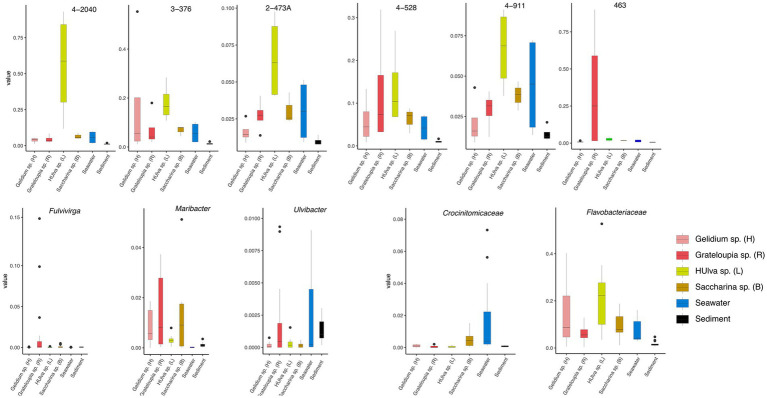
Boxplots show the RPKM values of strains 3-376^T^, 4-2040^T^, 2-473A^T^, 4-528^T^, 4-911^T^ and 463^T^. The relativate abundance of three genera of *Fulvivirga*, *Maribacter*, *Ulvibacter* and two families of *Crocinitomicaceae* and *Flavobacteriaceae*. The data was categorized based on different environmental sources or samples of *Gelidium* sp. (red algae), *Grateloupia* sp. (red algae), *Ulva* sp. (green algae), *Saccharina* sp. (brown algae), sediment and seawater.

This figure shows that the RPKM (Reads Per Kilobase per Million mapped reads) values of the strain 4-2040^T^, 3-376^T^, 2-473A^T^, 4-528^T^, 4-911^T^ and 463^T^ across different algal species (including *Gelidium* sp., *Grateloupia* sp., *Ulva* sp., and *Saccharina* sp.), seawater, and sediment samples (Figure 3). The results reveal that certain strains exhibit higher RPKM values in specific algal hosts, suggesting potential host-specific abundance. For example, 4-2040^T^, 3-376^T^, 2-473A^T^, 4-528^T^, 4-911^T^ have a higher RPKM values in *Ulva* sp. than others while the highest RPKM value of strain A463^T^ was in *Grateloupia* sp. In contrast, seawater and sediment samples generally show lower microbial abundance. Significant *p*-values (*p* < 0.05) further validate these differences, implying possible symbiotic relationships or ecological adaptations between algae and their associated microorganisms. These findings provide valuable insights into the functional roles and ecological dynamics of algal-associated microbial communities.

This figure illustrates the relative abundance of three bacterial genera (*Fulvivinga*, *Maribacter*, and *Ulvibacter*) across algal samples (*Gelidium* sp., *Ulva* sp., *Saccharina* sp.) and environmental samples (seawater, sediment) (Figure 3), along with their statistical characteristics. For *Fulvivirga*, the highest relative abundance was in *Grateloupia* sp. *Maribacter* has a high relative abundance in *Saccharina* sp. and *Grateloupia* sp. *Ulvibacter* was widely distributed across diverse algae (including *Grateloupia* sp.), seawater, and sediment. The figure presents statistical analyses comparing the relative abundances of two bacterial families, *Flavobacteriaceae* and *Crocinitomicaceae*, across distinct sample groups (Figure 3). The low *p*-value highlights statistically significant differences among groups, including *Gelidium* sp., *Grateloupia* sp., *Saccharina* sp., *Ulva* sp., seawater and sediment. For *Crocinitomicaceae*, the relative abundance in *Saccharina* sp. and seawater was relatively higher than others. For *Flavobacteriaceae*, the relative abundance in *Ulva* sp. was highest and in sediment was lowest. These results underscore the influence of environmental or host-specific factors on microbial community composition at the family level.

### 3.6 Putative polysaccharide degradative capacity

**Figure 4:**
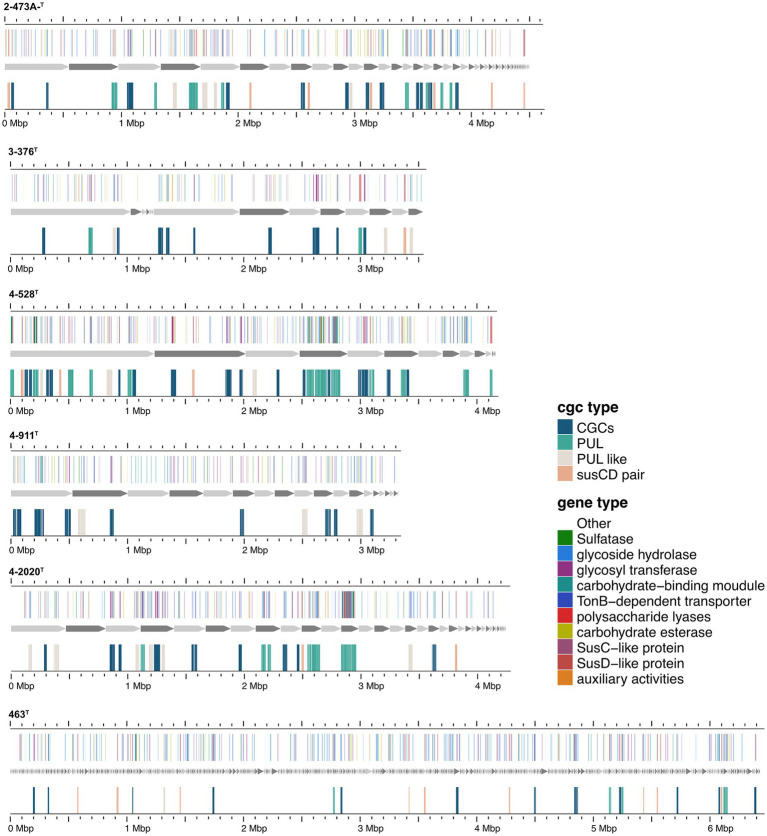
Composition and location of PUL, PUL-Like and CGCs on the genome of different strains. Strain names were labeled below each genome graphic. The bands from bottom to top of each strain indicated the type of polysaccharides degradation gene clusters, the composition of gene clusters, and the distribution location of its protein components.

The presence of degradative CAZymes and predicted PULs in each genome indicates the potential for polysaccharide degradation (Supplementary Table S3). Interestingly, the occurrence of PULs exhibited an exponential increase with the genome size, suggesting a correlation between genome size and polysaccharide-degrading capacity. Notably, *Cytophagaceae* species were annotated with a higher number of CAZymes and PULs, further underscoring their strong potential for polysaccharide degradation. *Flavobacteriaeae* species were annotated with a second higher number of CAZymes and PULs, *Crocinitomicaceae* species were annotated with fewest number of CAZymes and PULs. As shown in Figure 1, we observed a positive correlation between the numbers of CAZymes, sulfatases, and PULs and the different families of the phylogenetic tree. Specifically, the *Cytophagaceae* was annotated with a higher number of CAZymes, sulfatases, and PULs compared to the *Flavobacteriaeae* and *Crocinitomicaceae*. However, there were also exceptions. For example, the genome size of the newly discovered bacterium *Brumimicrobium ulvae* in our study was 3.3 Mb, which was similar to that of *Ulvibacter algarum* (3.5 Mb). However, *Brumimicrobium ulvae* contains 0 PULs (Figure 4), which was much smaller than the number of PULs in *Ulvibacter algarum*. The same results can be observed in other species within the genera to which these two bacteria belong. This discrepancy may be linked to specific environmental factors or ecological conditions related to its habitat.

**Figure 5:**
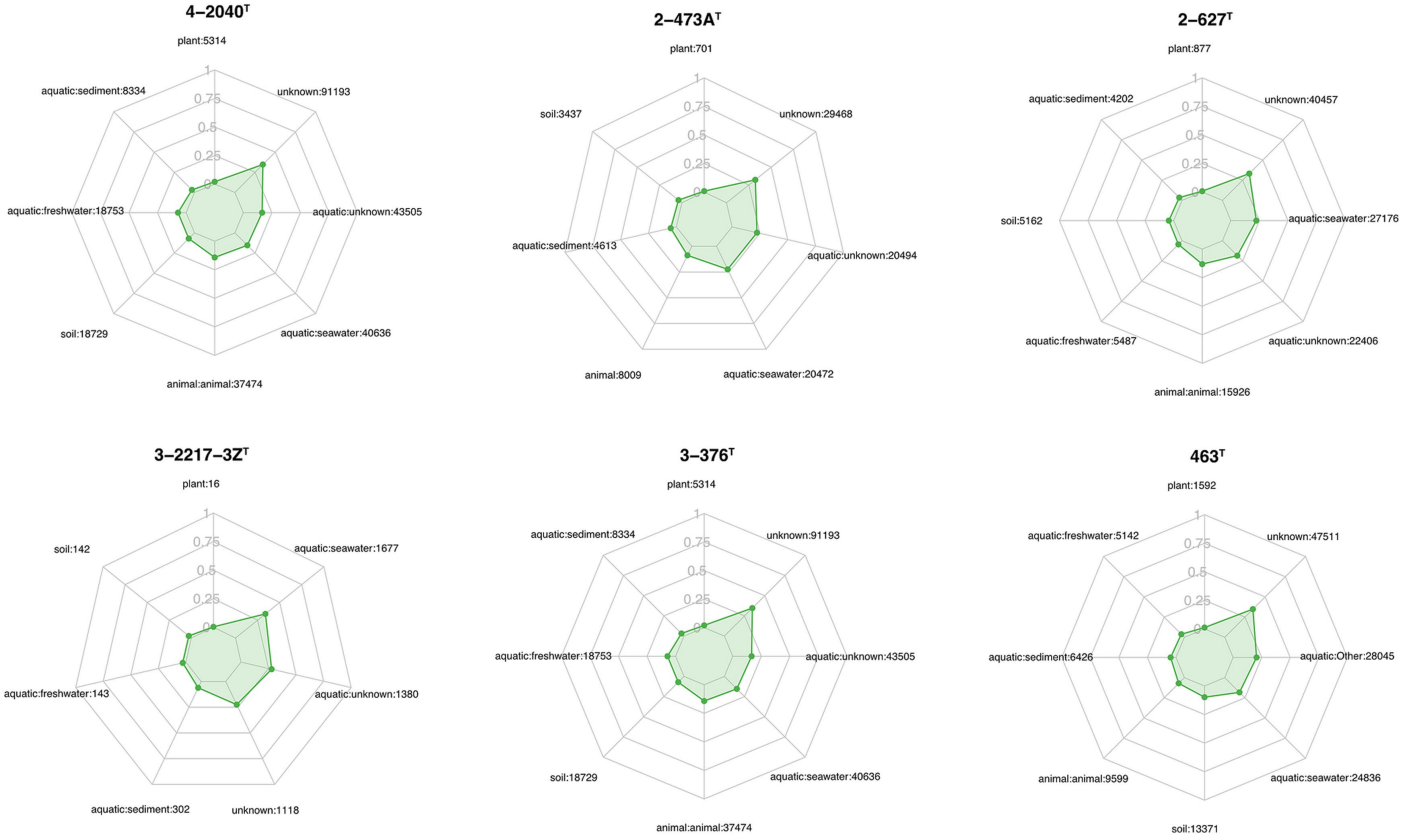
Ecological distribution of six strains across various habitats, as shown in the radar plots for different strains (3-376^T^, 4-2040^T^, 2-473A^T^, 4-528^T^, 4-911^T^ and 463^T^). Each plot illustrates the relative abundance of the strains across multiple environmental categories, including plant, aquatic freshwater, aquatic marine, aquatic sediment, animal habitats, aquatic unknown, soil, and unknown habitats. The numbers next to each habitat label represent the number of sequences identified within that category. The green shaded area in each radar plot indicates the strain’s distribution in that habitat, with the scale ranging from 0 to 1.

**Figure 6:**
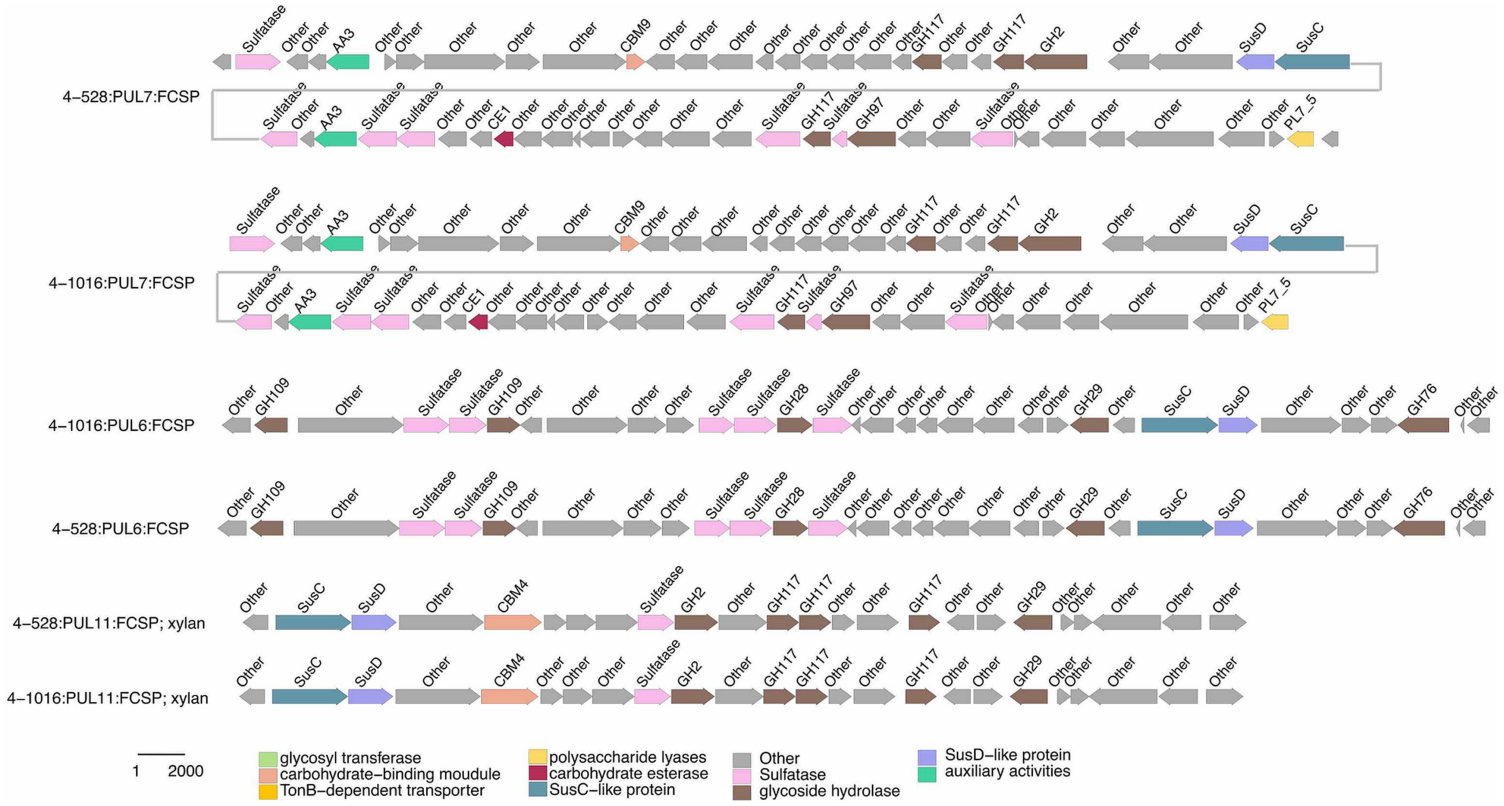
Selected PULs predicted to target substrates. Possible targets were FCSP.

Functional annotations revealed that strain 3-376^T^, 4-2040^T^, 2-473A^T^ and 4-528^T^ (*Flavobacteriaeae*) had 20, 90, 60, 88 CAZyme genes (Sum GH, PL, CE), and 4-911^T^ (*Crocinitomicaceae*) had 14 CAZyme genes, while strain 463^T^ contained 131 CAZymes. A list of CAZymes and its family activities in the genome of six strains were shown in Supplementary Table S7. Among these CAZymes, GHs were the greatest number of enzymes, which showed that they were more capable of degrading polysaccharides. The analysis of PULs across six bacterial strains revealed distinct substrate utilization profiles and metabolic versatility (Figures 4–6; Supplementary Figures S2–S5). Strain 4–528 exhibited the highest PUL diversity (11 PULs), targeting complex carbohydrates such as agar, alginate, fucosylated chondroitin sulfate (FCSP), and ulvan/rhamnan. 2-473A^T^ and 4-2040^T^ displayed moderate substrate ranges, including beta-mannan and laminarin/beta-glucan. In contrast, 463 and 3–376 showed limited PUL diversity, restricted to substrates like agar, starch, and galactomannan. Common substrates such as acetylxylan (targeted by 5 PULs) and alpha-glucan (4 PULs) highlighted functional redundancy across strains. Notably, multi-substrate PULs were identified, including 4-528^T^; PUL2 (degrading chondroitin sulfate, gellan, and hyaluronan) underscoring adaptive strategies for nutrient acquisition.

Functional comparisons further revealed conserved and divergent metabolic strategies: acetylxylan utilization was shared among 4-528^T^ and 2-473A^T^, while ulvan/rhamnan degradation was co-utilized by 4-528^T^ and 4-2040^T^. These patterns suggest both evolutionary conservation of key pathways and strain-specific adaptations to ecological niches. The findings emphasize the interplay between PUL diversity and substrate specificity, with implications for understanding microbial ecological roles and carbohydrate metabolism dynamics. A total of 189 annotated PULs can be linked to either dedicated polysaccharides or polysaccharide classes (Supplementary Table S8), and some of the larger PULs were attributed to multiple polysaccharide substrates. Due to differences in PUL composition, PULs with common substrate predictions were similar.

**Figure 7:**
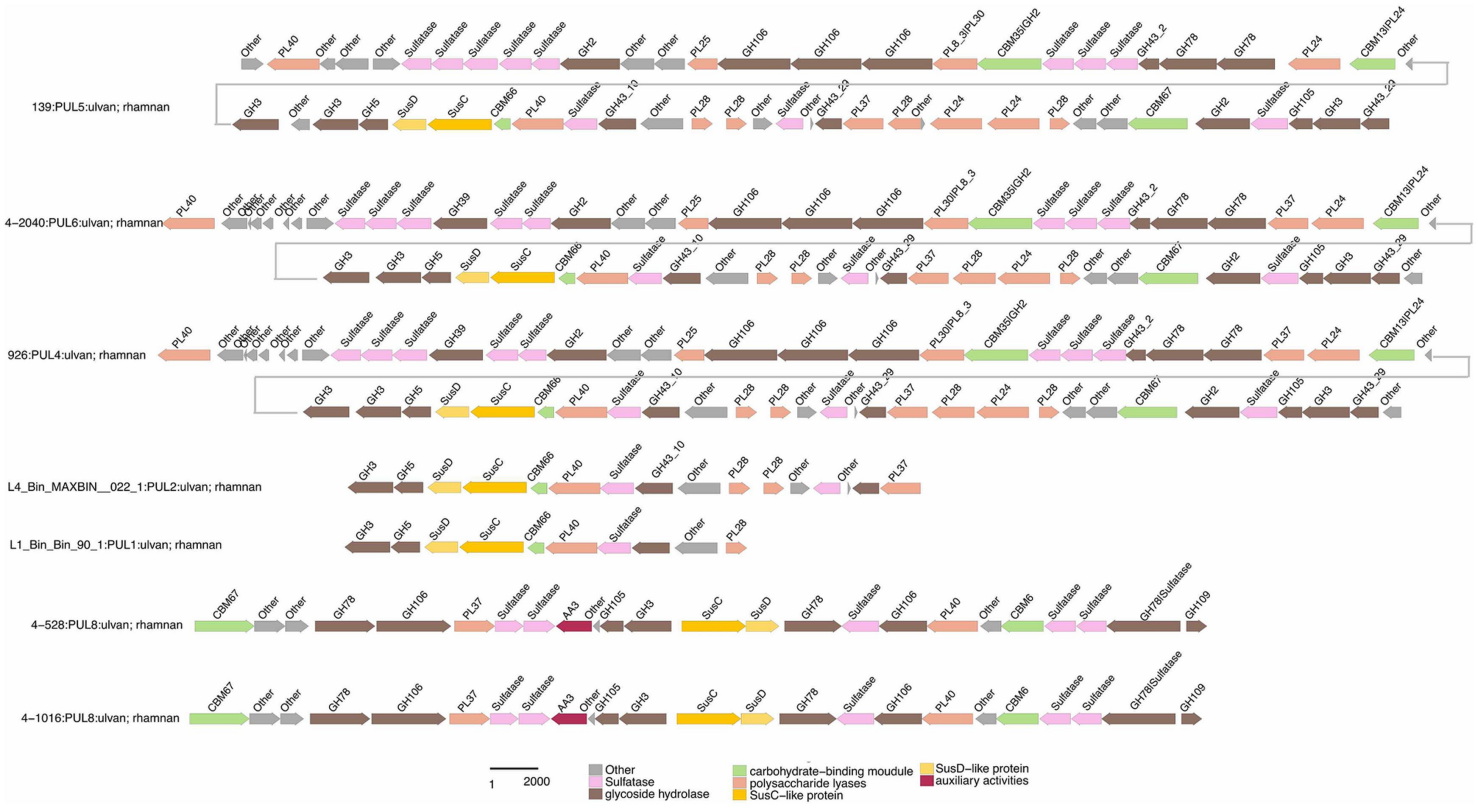
Selected PULs predicted to target substrates. Possible targets were ulvan/rhamnan.

**Figure 8:**
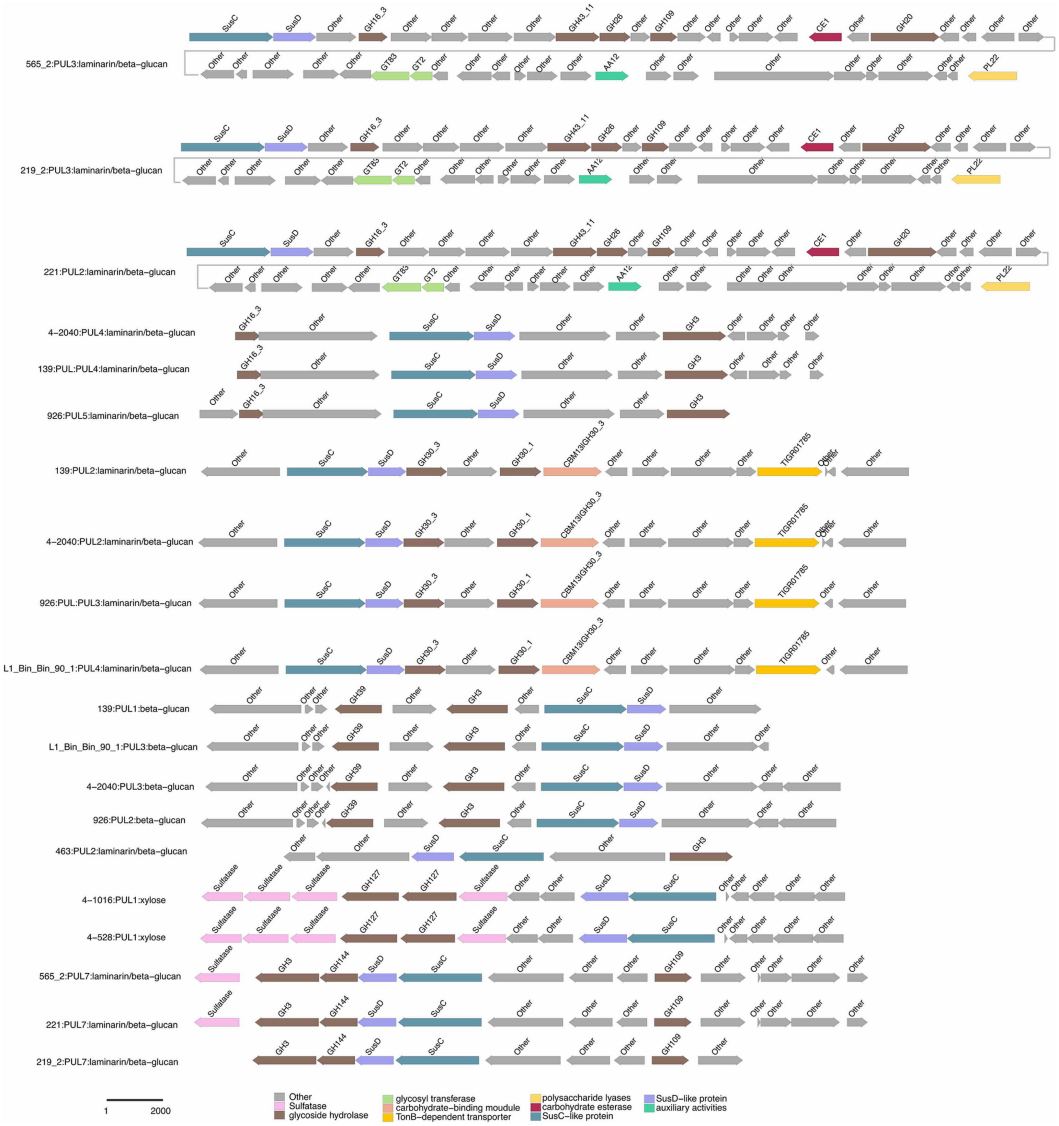
Selected PULs predicted to target substrates. Possible targets were laminarin/beta-glucan.

Specialized substrates exhibited narrower distributions. Fucosylated chondroitin sulfate (FCSP), a complex sulfated glycosaminoglycan (Sichert et al., 2020), was targeted by 4-528^T^; PUL6/PUL11, with 4-528^T^ dedicating two PULs to this niche substrate (Figure 6). The analysis of substrate-specific utilization across strains revealed distinct patterns of polysaccharide metabolism. For marine-associated carbohydrates, agar was uniquely targeted by 463^T^; PUL4 and 4-528^T^; PUL4, while ulvan/rhamnan was shared among 4-528^T^; PUL8 and 4-2040^T^; PUL1/PUL6 highlighting adaptation to algal-derived substrates. Alginate was metabolized by two strains (4-528^T^; PUL12, 3-376^T^; PUL2) (Figure 7), underscoring its relevance in marine or mucilaginous environments. Acetylxylan was the most widely targeted substrate (Supplementary Figure S2), with five PULs distributed among three strains (2-473A^T^; PUL1, and 4-528^T^; PUL1, PUL9, PUL10), indicating its ecological importance as a hemicellulose component. Similarly, alpha-glucan was utilized by four strains (4-528^T^; PUL5, 2-473A^T^; PUL4, 4-2040^T^; PUL5), with 4–528 and 4–2040 sharing PUL5 for starch/glycogen degradation, suggesting conserved metabolic strategies. Beta-glucan utilization was observed in three strains (4-2040^T^; PUL3, 2-473A^T^; PUL7), potentially reflecting roles in cellulose or laminarin breakdown. Laminarins were *β*-1,3-linked glucans that were abundant as they act as storage compounds in brown algae and diatoms. Enzymes from the GH families 5, 8, 9, 16, 17, 30, 64, 81, and 157 were involved in laminarin degradation (Krüger et al., 2019). The backbone was usually broken down by GH16_3 endo-glucanases. Just like the PUL structure (5) shown in the Figure 4, most strains can degrade laminarin and the PUL structure was relatively conserved. This reflects the fact that laminarin was one of the most abundant macromolecules in marine, as it acts as storage compound in brown macroalgae and diatoms. This type of PUL was highly prevalent in all strains (Figure 8).

Galactomannan (GH36) were strain-specific, limited to 2-473A^T^; PUL6, 463^T^; PUL1, and 2-473A^T^; PUL2, respectively. Starch utilization was shared by 463^T^; PUL3 and 2-473A^T^; PUL8. These patterns highlight functional redundancy for common substrates (e.g., acetylxylan, alpha-glucan) and strain-specific specialization for rare or complex carbohydrates (e.g., FCSP, chitobiose). Strain 4–528 emerged as the most versatile, targeting 11 substrates, compared to the narrower repertoires of 463^T^ and 3-376^T^. This substrate-centric analysis underscores the interplay between ecological adaptation and metabolic diversity in polysaccharide utilization.

Chondroitin sulfate utilization reveals multi-substrate metabolic strategies (4–528; PUL2, 4–2040; PUL1). These patterns underscore the ecological relevance of sulfated glycosaminoglycan metabolism in marine or host-associated environments and highlight the functional plasticity of PULs in adapting to structurally complex carbohydrates. Interestingly, they possess two distinct SusC/SusD gene pairs (Figure 5). Alginates were linear co-polymers consisting of homopolymeric blocks of (1 → 4)-linked β-D-mannuronate and *α*-L-guluronate residues that were covalently linked in alternating sequences or blocks (Draget and Taylor, 2011). Alginate utilization highlights metabolic diversity among strains: from single-substrate specialization (4–528; PUL12) to multifunctional strategies (3–376; PUL2). These differences reflect adaptive evolution to distinct ecological niches, such as marine environments for algal polysaccharide specialists or host-associated habitats for generalist strains. This analysis provides molecular insights into microbial carbon cycling and informs applications in alginate biodegradation technologies. Alginate PULs encode PL6, 7, 14, 15, and 17 family alginate lyases (Rønne et al., 2023). Alginate, unlike laminarin, shows small variation across different strains in difference species.

## 4 Conclusion and discussion

Marine ecosystems rely critically on polysaccharide degradation mediated by microorganisms, particularly members of the phylum *Bacteroidota* laminarin (Avcı et al., 2020), which play a pivotal role in global carbon cycling. Elucidating the enzymatic and metabolic pathways these microbes employ to break down complex carbohydrates was essential for advancing mechanistic understanding of microbial decomposition processes and informing sustainable ocean resource management strategies (Sichert et al., 2020). Our research identified six novel *Bacteroidota* species belong to *Flavobacteriaeae*, *Cytophagaceae* and *Crocinitomicaceae* isolated from marine macroalgae and conducted a thorough phylogenetic, genomic and phenotypic analysis. Subsequently, by analyzing genome-wide, we conducted a basic comparative analysis of the genomic features and metabolic potential of *Bacteroidota* strains.

We then focused on analyzing the similarities and differences among *Flavobacteriaeae*, *Cytophagaceae* and *Crocinitomicaceae* strains in terms of their polysaccharide degradation abilities for different polysaccharide substrates. Additionally, we observed a positive correlation between the genome size and the number of PULs (Avcı et al., 2020), suggesting that strains with larger genomes may possess a stronger polysaccharide degradation capacity (Figure 1). We observed significant diversity in polysaccharide degradation capabilities among the species of *Flavobacteriaeae* and *Cytophagaceae*, with apparent direct correlation to their taxonomy. Within the same genus, strains exhibited similar PUL profiles and genome sizes. These results suggest that the composition of a species’ PUL repertoire was influenced more by its phylogenetic lineage.

We observed diverse polysaccharide degradation capacities among *Cytophagaceae* strains, such as for laminarin and alginate. Through comparative analysis, we speculate the presence of PULs involved in the degradation of laminarin and alginate. Alginate was a polysaccharide, known for its solubility and gel-forming properties. Laminarin, found in brown algae, shares structural similarities with alginate but has distinct sugar units (Becker et al., 2020). Both polysaccharides play crucial roles in the structure of algal cell walls and act as carbon sources for marine microorganisms. Such as laminarin was a major molecule in the marine carbon cycle (Becker et al., 2020). The PUL repertoires of the isolates reveal that common and structurally simple polysaccharides, such as laminarin, *α*-1,4-glucans, and alginate, were frequently targeted by conserved PULs (Figure 8; Supplementary Figure S3). This suggests that maintaining the enzymatic machinery for degrading these substrates was advantageous for marine *Flavobacteriaeae* (Mann et al., 2013; Alejandre-Colomo et al., 2021; Lee et al., 2023) and *Cytophagaceae* (Ma et al., 2025). This convergence in PUL composition was attributed to the core microbial communities on macroalgal surfaces, which have evolved PULs capable of degrading diverse macroalgal polysaccharides as an adaptive strategy for colonization across various macroalgal species (Lu et al., 2023). These data revealed that epiphytic bacteria derived from different macroalgae and isolation sources possess similar PULs for degrading identical polysaccharides (Figures 6, 7; Supplementary Tables S7, S8). For example, both 3-376^T^ and 2-473A^T^ were involved in acetylxylan (Figure 6; Supplementary Table S8). Such functional adaptation enhances their ability to thrive in the unique environmental niches provided by macroalgal surfaces. Furthermore, the variability in bacterial polysaccharide-degrading capabilities reflects their specificity and diversity in metabolizing polysaccharide substrates (McKee et al., 2021). Certain bacteria may specialize in degrading one or a few specific polysaccharides, while others exhibit a broader degradative capacity (Figures 6, 7, Supplementary Tables S7, S8). For instance, strain 3-376^T^ may degrade alginate and α-glucan (Supplementary Figure S3), 463^T^ may degrade agar (Supplementary Figure S4), while 2-473A^T^ may degrade galactomannan (Supplementary Figure S5).

The functional predictions of PULs in this study primarily rely on sequence similarity-based bioinformatics approaches. While these methods provide valuable insights, their resolution remains inherently lower than that of direct experimental validation. Current limitations in characterizing marine macroalgal polysaccharides may introduce uncertainties in substrate specificity predictions. Nevertheless, our systematic profiling of PUL distributions across a phylogenetically diverse collection of isolates from a shared habitat enables the identification of ecologically recurrent PUL modules with high biological relevance. These conserved PULs serve as priority targets for functional characterization and provide a robust framework for formulating testable hypotheses about their putative polysaccharide substrates. Despite its constraints, this strategy establishes a novel paradigm for prioritizing environmentally significant polysaccharides that were recalcitrant to conventional biochemical analyses. Future investigations should integrate genome-guided metabolomics and enzymatic activity assays to elucidate the ecological contributions of these bacterial lineages and assess their biotechnological potential in marine carbon cycling and algal biomass valorization.

## Description of *Eudoraea algarum* sp. nov.

*Eudoraea algarum* (al.Ga’rum. L. Gen. Fem. Pl. n. *Algarum*, of/from algae).

Cells are Gram-stain-negative, strictly aerobic and rod-shaped, 2.1–2.4 μm in length and 0.2–0.3 μm in width. Colonies on MA are yellow, circular and opaque with entire edges after 2–3days of cultivation. Growth is observed at between 20 and 35°C (optimum, 30–33°C), pH between 6.0 and 9.0 (optimum, pH 7.5) and in the presence of 1–6% (w/v) NaCl (optimum, 3–4%). Nitrate is not reduced to nitrite. Positive for catalase activity and hydrolysis of Tween 20, starch, DNA and casein, negative for oxidase activity and hydrolysis of Tweens 40, 60 and 80, gelatin and cellulose. *β*-glucosidase (aesculin hydrolysis) and β-galactosidase (PNPG) are positive, but negative reactions for indole production, glucose fermentation, arginine dihydrolase, urease, protease (gelatin hydrolysis) and for all tested assimilation substrates, including glucose, arabinose, mannitol, maltose, potassium gluconate, mannose, N-acetyl-glucosamine, caprate, adipate, malate, citrate and phenylacetate. The major fatty acids are iso-C_15: 0_, iso-C_15: 1_ G, and iso-C_17: 0_ 3-OH when grown at 28°C. Contains MK-6 as the only respiratory quinone. The predominant polar lipids are phosphatidylethanolamine, aminolipid and unidentified lipid.

The type strain is 2-473A^T^ (= MCCC 1H00698^T^ = KCTC 102410^T^), was isolated from red maroalgae in marine (*Gelidium* sp.) (122.12 N, 37.56 E). The DNA G + C content of the type strain is 45.1 mol%.

## Description of *Maribacter algarum* sp. nov.

*Maribacter algarum* (al.Ga’rum. L. Gen. Fem. Pl. n. *Algarum*, of/from algae).

Cells are Gram-stain-negative, facultative anaerobic and rod-shaped, 0.8–1.0 μm in length and 0.3 μm in width. Colonies on MA are yellow, circular and opaque with entire edges after 2–3days of cultivation. Growth is observed at between 10 and 40°C (optimum, 25–30°C), pH between 5.5 and 9.0 (optimum, pH 8.0) and in the presence of 0–10% (w/v) NaCl (optimum, 2–5%). Nitrate is reduced to nitrite. Positive for oxidase and catalase activity and hydrolysis of gelatin, Tween 20, 40, 60 and 80 and starch, negative for hydrolysis of cellulose, DNA and casein. Cells exhibited *β*-glucosidase activity but tested negative for L-tryptophan utilization, D-glucose fermentation, arginine dihydrolase, urease, β-galactosidase, gelatin liquefaction, glucose fermentation, arabinose fermentation, mannose fermentation, mannitol fermentation, N-acetylglucosamine fermentation, maltose fermentation, potassium gluconate utilization, capric acid oxidation, adipic acid oxidation, malic acid oxidation, citrate utilization, and phenylacetic acid oxidation. The major fatty acids are iso-C_15: 0_, iso-C_15: 1_ G, and iso-C_17: 0_ 3-OH when grown at 28°C. Contains MK-6 as the only respiratory quinone. The predominant polar lipids are phosphatidylethanolamine, and phospholipid.

The type strain is 4-528^T^ (= MCCC 1H00803^T^ = KCTC 102405^T^), was isolated from red maroalgae in marine (*Gelidium* sp.) (122.12 N, 37.56 E). The DNA G + C content of the type strain is 35.2 mol%.

## Description of *Brumimicrobium ulvae* sp. nov.

*Brumimicrobium ulvae* (ul’vae. L. Gen. Fem. n. *Ulvae*, of a seaweed).

Cells are Gram-stain-negative, strictly aerobic and rod-shaped, 1.3–1.7 μm in length and 0.2 μm in width. Colonies on MA are orange, circular and opaque with entire edges after 2–3days of cultivation. Growth is observed at between 10 and 35°C (optimum, 25–28°C), pH between 5.5 and 9.0 (optimum, pH 7.5) and in the presence of 1–8% (w/v) NaCl (optimum, 6%). Nitrate is not reduced to nitrite. Positive for oxidase and catalase activity and hydrolysis of gelatin, Tween 40, 60 and 80, casein, DNA, and cellulose, negative for hydrolysis of Tween 20, and starch. Cells exhibited *β*-glucosidase activity but tested negative for L-tryptophan utilization, D-glucose fermentation, arginine dihydrolase, urease, β-galactosidase, gelatin liquefaction, glucose fermentation, arabinose fermentation, mannose fermentation, mannitol fermentation, N-acetylglucosamine fermentation, maltose fermentation, potassium gluconate utilization, capric acid oxidation, adipic acid oxidation, malic acid oxidation, citrate utilization, and phenylacetic acid oxidation. The major fatty acids are iso-C_15: 0_ and iso-C_15: 1_ G when grown at 28°C. Contains MK-6 as the only respiratory quinone. The predominant polar lipids are phosphatidylethanolamine, and aminolipid.

The type strain is 4-911^T^ (= MCCC 1H00873^T^ = KCTC 102404^T^), was isolated from red maroalgae in marine (*Ulva* sp.) (122.12 N, 37.56 E). The DNA G + C content of the type strain is 36.1 mol%.

## Description of *Ulvibacter algarum* sp. nov.

*Ulvibacter algarum* (al.Ga’rum. L. Gen. Fem. Pl. n. *Algarum*, of/from algae).

Cells are Gram-stain-negative, strictly aerobic and ovoid-shaped, 0.4–0.9 μm in length and 0.3–0.4 μm in width. Growth is observed at between 10 and 37°C (optimum, 25°C), pH between 5.5 and 9.0 (optimum, pH 7.0–8.0) and in the presence of 0–6% (w/v) NaCl (optimum, 1–3%). Nitrate is not reduced to nitrite. Oxidase and catalase activities are present. Tweens 20, 40, 60 and 80 are decomposed. Gelatin, agar, casein, starch, DNA and cellulose are not hydrolyzed. Cells exhibited weak *β*-glucosidase activity but tested negative for all other enzymatic and metabolic traits assessed, including L-tryptophan utilization, D-glucose fermentation, arginine dihydrolase, urease, β-galactosidase, gelatin liquefaction, glucose fermentation, arabinose fermentation, mannose fermentation, mannitol fermentation, N-acetylglucosamine fermentation, maltose fermentation, potassium gluconate utilization, capric acid oxidation, adipic acid oxidation, malic acid oxidation, citrate utilization, and phenylacetic acid oxidation. The major fatty acids are iso-C_15: 0_, iso-C_17: 0_ 3-OH and summed feature 3 (comprising C_16: 1_*ω6c* and/or C_16: 1_*ω7c*) when grown at 28°C. Contains MK-6 as the only respiratory quinone. The predominant polar lipids are phosphatidylethanolamine and aminolipid.

The type strain is 3-376^T^ (= MCCC 1H01507^T^), isolated from red maroalgae in marine (*Gelidium* sp.) (122.12 N, 37.56 E). The DNA G + C content of the type strain is 34.4 mol%.

## Description of *Tamlana algarum* sp. nov.

*Tamlana algarum* (al.Ga’rum. L. Gen. Fem. Pl. n. *Algarum*, of/from algae).

Cells are Gram-stain-negative, facultative anaerobic and spherical-shaped, 0.4 μm in length and 0.4 μm in width. Colonies on MA are yellow, circular and opaque with entire edges after 2–3days of cultivation. Growth is observed at between 15 and 35°C (optimum, 28°C), pH between 6.0 and 9.0 (optimum, pH 7.5) and in the presence of 1–6% (w/v) NaCl (optimum, 3%). Nitrate is reduced to nitrite. Positive for oxidase and catalase activity and hydrolysis of Tweens 20, 40 and 60, negative for oxidase activity and hydrolysis of Tweens 80, starch, casein, gelatin, DNA and cellulose. Cells exhibited *β*-glucosidase activity but tested negative for L-tryptophan utilization, D-glucose fermentation, arginine dihydrolase, urease, β-galactosidase, gelatin liquefaction, glucose fermentation, arabinose fermentation, mannose fermentation, mannitol fermentation, N-acetylglucosamine fermentation, maltose fermentation, potassium gluconate utilization, capric acid oxidation, adipic acid oxidation, malic acid oxidation, citrate utilization, and phenylacetic acid oxidation. The major fatty acids are iso-C_15: 0_, iso-C_15: 1_ G, iso-C_15: 0_ 3-OH and iso-C_17: 0_ 3-OH when grown at 28°C. Contains MK-6 as the only respiratory quinone. The predominant polar lipids are phosphatidylethanolamine, aminolipids and unidentified lipid.

The type strain is 4-2040^T^ (= MCCC 1H00922^T^), isolated from brown maroalgae in marine (*Saccharina* sp.) (122.12 N, 37.56 E). The DNA G + C content of the type strain is 35.4 mol%.

## Description of *Aurantiphycus algarum* sp. nov.

*Aurantiphycus algarum* (al.Ga’rum. L. Gen. Fem. Pl. n. *Algarum*, of/from algae).

Cells are Gram-stain-negative, facultative anaerobic, and ovoid-shaped, 0.5–0.6 μm in length and 0.3–0.4 μm in width. Colonies on MA are orange, circular and opaque with entire edges after 2–3days of cultivation. Growth is observed at between 20 and 35°C (optimum, 28°C), pH between 5.5 and 9.0 (optimum, pH 7.5) and in the presence of 0–6% (w/v) NaCl (optimum, 2–3%). Nitrate is reduced to nitrite. Positive for oxidase activity and hydrolysis of starch, casein and DNA, negative for catalase activity and hydrolysis of Tween 20, 40, 60 and 80, gelatin, and cellulose. Cells exhibited β-glucosidase and β-galactosidase, activity but tested negative for L-tryptophan utilization, D-glucose fermentation, arginine dihydrolase, urease, glucose fermentation, arabinose fermentation, mannose fermentation, mannitol fermentation, N-acetylglucosamine fermentation, maltose fermentation, potassium gluconate utilization, capric acid oxidation, adipic acid oxidation, malic acid oxidation, citrate utilization, and phenylacetic acid oxidation. The major fatty acids are iso-C_15: 0_, iso-C_15: 1_ G, iso-C_17: 0_ 3-OH, summed feature 8 (comprising C_16: 1_*ω6c* and/or C_16: 1_*ω7c*) when grown at 28°C. Contains MK-7 as the only respiratory quinone. The predominant polar lipids are phosphatidylethanolamine, aminolipid and unidentified lipid.

The type strain is 463^T^ (= MCCC 1H00865^T^), was isolated from red maroalgae in marine (*Grateloupia* sp.) (122.12 N, 37.56 E). The DNA G + C content of the type strain is 43.9 mol%.

## Data Availability

The GenBank accession number for the 16S rRNA gene sequence of strain 3-376^T^, 4-2040^T^, 2-473A^T^, 4-528^T^, 4-911^T^ and 463^T^ were PV684998, PV685000, PV685001, PV685002, PV684997 and PV684999 respectively. The draft genome of strain 3-376^T^, 4-2040^T^, 2-473A^T^, 4-528^T^, 4-911^T^ and 463^T^ had been deposited in GenBank under the accession number CANLBJ000000000, CANMAQ000000000, JBNGOQ000000000, CANMAW000000000, CANNGT000000000 and CANLOR000000000 respectively. BioSample: SAMEA112156868, SAMN40544020; BioProject: PRJEB57783. Sequences were available from the European Nucleotide Archive under accessions PRJEB50838 (metagenomes and MAGs), and PRJEB57783 (genomes of cultured bacteria).

## Footnotes

^1^ www.ezbiocloud.net/tools/contest16s

^2^ https://blast.ncbi.nlm.nih.gov

^3^ http://www.ezbiocloud.net

^4^ http://www.cazy.org/

^5^ https://github.com/widdowquinn/pyani

^6^ https://github.com/2015qyliang/POCP
