# Supplementary material for: Six new bacterial species isolated from the phycosphere of marine macroalgae: a joint analysis based on taxonomy and polysaccharide utilization loci
Source: Front Microbiol. 2025 Jul 18;16:1642517. doi: 10.3389/fmicb.2025.1642517 (PMC12313589; doi:10.3389/fmicb.2025.1642517)
Supplement: Supplementary file 1 [file Supplementary_file_1.docx]

Supplementary Material

**Discovery of six new species in the core families of marine macroalgae: a joint analysis based on taxonomy and polysaccharide utilization loci**

Dechen Lu^1,2†^, Yuan Ying^1†^, Xin-Yun Tan^2^, Le Liu^2^, Jin-Hao Teng^1^, Hai-Yan Cong^2^, Xue Cui^2^, Tian-He Liu^1,2^, Jing Zhang^2^, Zong-Jun Du^1,2*^ and Ming-Yi Wang^2*^

^1^Weihai Municipal Hospital, Weihai, 264209, China

^2^Marine College, Shandong University, Weihai, Shandong, 264209, China

*** Correspondence:**

Zong-Jun Du, duzongjun@sdu.edu.cn;

Ming-Yi Wang, wangmingyi1973@outlook.com

# Supplementary Figures

**Supplementary Fig. S1.** Two-dimensional TLC plate images of lipids of strain 4-528^T^, 2-473A^T^, 4-911^T^, 3-376^T^, 463^T^ and 4-2040^T^. The plate is sprayed with 5% molybdatophosphoric acid to show all lipids. L1-4, Lipid; PL, Phospholipid; PE, Phosphatidylethanolamine.


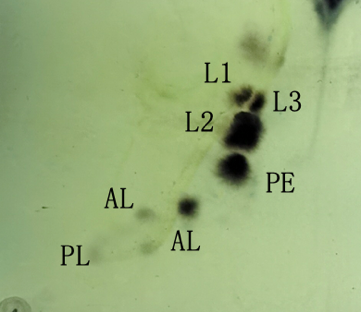

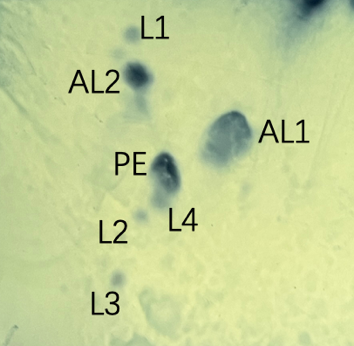

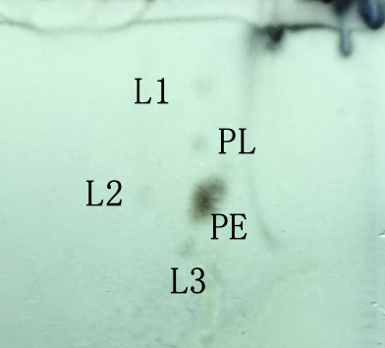
4-528^T^ 2-473A ^T^ 4-911^T^

**Second dimension**

**First dimension**

**
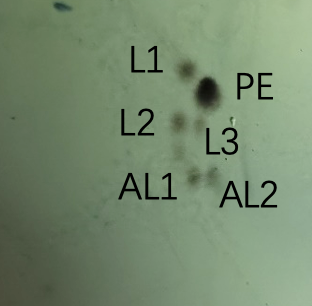
**
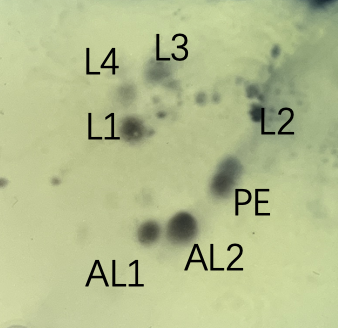

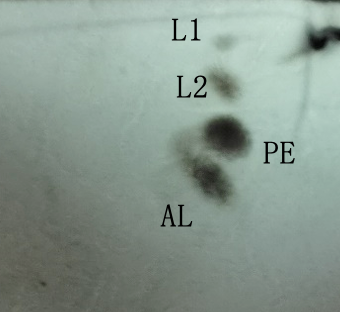
 3-376^T^ 463^T^ 4-2040^T^

**Second dimension**

**First dimension**

**Supplementary Fig. S2.** Selected PULs predicted to target substrates. Possible targets are acetylxylan/beta-mannan.

**Supplementary Fig. S3.** Selected PULs predicted to target substrates. Possible targets are alginate.

**Supplementary Fig. S4.** Selected PULs predicted to target substrates. Possible targets are agar.

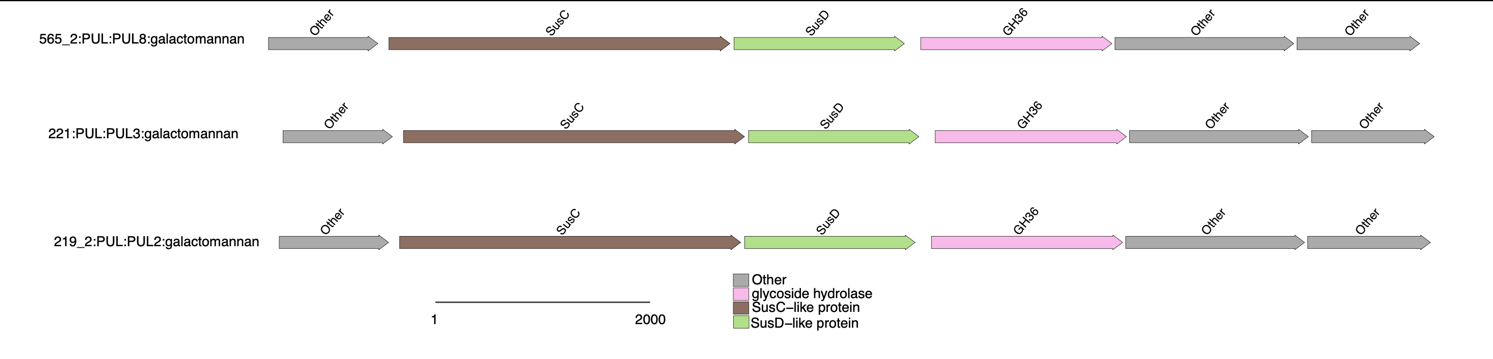
**Supplementary Fig. S5.** Selected PULs predicted to target substrates. Possible targets are galactomannan.
